# Time for a More Precise and Practical Laboratory Definition of Metainflammation in Obesity-Related Diseases

**DOI:** 10.3390/medsci14020303

**Published:** 2026-06-11

**Authors:** Ivica Petrović, Miloš N. Milosavljević, Ana V. Pejčić

**Affiliations:** 1 Department of Pathophysiology, Faculty of Medical Sciences, University of Kragujevac, 34000 Kragujevac, Serbia; ivica.petrovic@fmn.kg.ac.rs; 2 Department of Pharmacology and Toxicology, Faculty of Medical Sciences, University of Kragujevac, 34000 Kragujevac, Serbia; anapejcic201502@yahoo.com

**Keywords:** metainflammation, low-grade inflammation, metabolic syndrome, cardiovascular risk, thrombosis

## Abstract

**Background/Objectives**: Metainflammation is a chronic low-grade inflammatory state driven by excessive nutrition and obesity. It is associated with adipocyte dysfunction, altered adipokine secretion, and the development of insulin resistance, which contributes to dysglycemia and hyperglycemia. Recent initiatives emphasize earlier recognition of pathological processes to enable timely detection and primary prevention of adverse cardiovascular outcomes. Metainflammation has traditionally been assessed using serum C-reactive protein (CRP) levels and several proposed indices, with inconsistent performance due to chronic inflammation-related changes in blood cell counts. This study aimed to evaluate a newly proposed MetaLGI score as a laboratory-based tool for identifying pronounced metainflammation in patients with metabolic syndrome. **Methods**: Patients with metabolic syndrome were assessed for metainflammation using the newly proposed MetaLGI score and compared with previously suggested indices and CRP-based definitions. The ability of these approaches to identify pronounced metainflammation and associated hematological and metabolic alterations was evaluated. **Results**: The MetaLGI score demonstrated superiority in recognizing pronounced metainflammation in patients with metabolic syndrome compared to previous indices. Additionally, the proposed laboratory definition of metainflammation showed better performance in identifying patients with increased platelet counts, highlighting its relevance for inflammation-related cardiovascular thrombosis. MetaLGI also outperformed earlier proposals in identifying patients with increased β-cell burden. **Conclusions**: The newly proposed MetaLGI score represents a superior and widely available laboratory-based method for detecting pronounced metainflammation in patients with metabolic syndrome. Its improved ability to identify increased platelet counts and β-cell burden suggests potential value for early risk stratification and primary prevention of cardiometabolic complications.

## 1. Introduction

Metabolic syndrome (MetS) is a disorder characterized by the presence of at least three of five metabolic abnormalities, which include central obesity, insulin resistance, dysglycemia, atherogenic dyslipidemia, and hypertension, and is a midpoint on a continuum of obesity-related diseases [1]. The pathophysiological mechanism that leads to the development of MetS is complex and integrates processes such as the increase in visceral adipose tissue associated with adipocyte dysfunction, the development of metainflammation and the appearance of insulin resistance [2].

Metainflammation is chronic low-grade systemic inflammation, metabolically caused, and is characterized by increased levels of various pro-inflammatory mediators, which could be observed as biomarkers of metainflammation [3,4]. Among the aforementioned biomarkers of metainflammation, the most notable is the C-reactive protein (CRP), which is known to be elevated in patients with MetS [5]. Metainflammation in patients with MetS is active in all tissues involved in energy homeostasis, and it is believed that macrophages play a significant role in its development [6]. Excessive activation of macrophages results in increased synthesis and secretion of pro-inflammatory cytokines, primarily interleukin 6 (IL-6), tumor necrosis factor, and interferon gamma, in concentrations which are elevated in patients with MetS [2,3,6]. From a pathophysiological perspective, the infiltration of adipose tissue by monocytes, and their activation and transformation into macrophages with increased production of pro-inflammatory citokines, leads to increased production of CRP and fibrinogen [7]. Finally, we must not overlook the increasing role that platelets play in the process of coordination and amplification of inflammation and/or metainflammation, which is particularly important in the pathogenesis of cardiovascular events in patients with MetS and type 2 diabetes [8]. This phenomenon is reflected in the well-known enhanced CD40/CD40L interaction in platelets in a pro-inflammatory environment, the release of pro-inflammatory platelet-derived microparticles, and the excretion rate of the major enzymatic metabolite thromboxane [9,10,11]. Also, we should not neglect the formation of platelet–leukocyte aggregates, predominantly with neutrophils and monocytes, which play an important role in orchestrating the innate immune response [12,13]. Platelets also express functional TLR2 and TLR4, whose activation by lipopolysaccharide further alters platelet–leukocyte interactions and platelet hyperreactivity [14]. Platelets have been shown to activate and recruit leukocytes to sites of infection and inflammation, and to modulate leukocyte behavior [15]. All of these effects trigger a series of events occurring in the vascular wall and in the circulation during the ongoing inflammatory response, and are particularly enhanced in patients with MetS [16,17]. Yudkin et al. showed that there is a strong correlation between markers of inflammation and individual features of the MetS [18]. Based on this knowledge, a special predictive score, such as the systemic inflammation response index (SIRI), has been designed to predict all-cause mortality and cardiovascular mortality [19].

The aim of this study was to evaluate the discriminative performance of commonly available inflammatory biomarkers in identifying pronounced metainflammation in patients with MetS, and to propose a novel index—the Meta Low-Grade Inflammation (MetaLGI) score—as a potentially more accurate and accessible tool for assessing the degree of metainflammation compared to existing definitions based on CRP levels and the SIRI, in a study with a cross-sectional design.

## 2. Materials and Methods

An observational, non-invasive, cross-sectional study was conducted on patients referred to the daily hospital of the Center for Endocrinology, Diabetes and Metabolic Diseases at the University Clinical Center Kragujevac (Kragujevac, Serbia). To minimize potential selection bias, all consecutive patients referred to the daily hospital of the Center for Endocrinology, Diabetes and Metabolic Diseases during the study period who met the eligibility criteria were invited to participate. We included patients older than 18 years who signed an informed consent form. In order to minimize potential confounding factors that could influence inflammatory and metabolic parameters, strict exclusion criteria were applied. Exclusion criteria were as follows: a previous diagnosis of diabetes; use of oral anti-diabetic drugs, obesity medications, glucocorticoids, immunomodulatory drugs, antipsychotics, or antidepressants; an acute infection within the past two weeks; a history of malignant disease in the past five years; liver or kidney insufficiency; chronic autoimmune diseases; recent surgery or major trauma; pregnancy; and active cardiovascular events. Participants were divided into two groups: one group of patients with MetS, according to the International Diabetes Federation classification, and a control group, which included healthy subjects. The following data were collected: age, gender, waist circumference, triglycerides, high-density lipoprotein cholesterol (HDL), systolic and diastolic blood pressure, IL-6, CRP, fibrinogen, white blood cell count (WBC), absolute neutrophil count (ANC), absolute lymphocyte count (ALC), absolute monocyte count (AMC), platelet count (PLT), and percentage of neutrophils, lymphocytes, monocytes, aspartate aminotransferase (AST), and alanine aminotransferase (ALT). The Homeostatic Model Assessment for Insulin Resistance (HOMA-IR), Homeostasis Model Assessment of β-cell function (HOMA-B) and neutrophil-to-lymphocyte ratio (NLR) were calculated. Venous blood samples for laboratory testing were collected in the morning after 12 h of overnight fasting. The study was conducted in accordance with the Declaration of Helsinki, and approved by the Ethics Committee of the University Clinical Centre Kragujevac prior to its initiation (number 01/22-432, date 5 December 2022).

In our research, to estimate the degree of metainflammation, in addition to the high normal CRP concentration (>2.5 mg/L), we also used the SIRI, which is calculated by the following formula: (Neutrophil count × Monocyte count)/Lymphocyte count [10]. Pronounced metainflammation was defined by an SIRI score > 1.35 × 109.

We defined a new index, which we named the MetaLGI score, which includes the assessment of CRP concentration (>2.5 mg/L) and fibrinogen (>3.0 g/L), monocyte (>0.55 × 10^9^/L), and platelet counts (>250 × 10^9^/L), as widespread, accessible, and inexpensive parameters that simultaneously speak in favor of the degree of metainflammation. Pronounced metainflammation was defined by the MetaLGI score as the simultaneous presence of CRP, fibrinogen, and monocyte and platelet counts above the previously defined values. We defined the MetaLGI score as a binary index. A subject receives a positive MetaLGI score when all four inflammatory parameters are simultaneously above the predefined thresholds (CRP > 2.5 mg/L, fibrinogen > 3.0 g/L, monocyte count > 0.55 × 10^9^/L, and platelet count > 250 × 10^9^/L). In all other cases, the score is classified as negative.

This study was designed as an exploratory cross-sectional study. An a priori sample size calculation was performed using G*Power software (version 3.1.9.2) based on differences in CRP levels reported in a previous study that included three groups of participants: a control group and two MetS groups stratified according to the platelet/HDL ratio as an indicator of disease severity [20,21]. Sample size estimation was conducted using the F-test family (ANOVA: Fixed effects, omnibus, one-way), assuming α error probability of 0.05, statistical power of 0.80, three study groups, and using an effect size of f = 0.737 calculated based on the reported CRP data in the reference study. The analysis indicated a minimum required total sample size of 24 participants. The final sample consisted of 60 participants (37 patients with MetS and 23 controls).

The Statistical Program for Social Sciences (SPSS version 22) was used for the statistical analysis. Data were analyzed using descriptive statistics. The Shapiro–Wilk test was applied to assess the normality of distribution of continuous variables. For normally distributed data, an independent-samples *t*-test (for two groups) and one-way analysis of variance (ANOVA, for three groups) were used to compare differences between groups. For non-normally distributed data, we used the Kruskal–Wallis test (for three groups) and Mann–Whitney U test (for two groups). Pairwise group comparisons were performed using independent-samples *t*-tests or Mann–Whitney U tests, as appropriate, with Bonferroni correction applied for multiple post hoc comparisons. The χ^2^ test was used to compare categorical variables. A *p*-value of <0.05 was considered statistically significant, except in analyses with three comparisons, where the Bonferroni correction was applied, and a *p*-value of <0.017 was considered statistically significant. The ROC curve was used to analyze the prognostic values of serum IL-6. A 95% confidence interval was used for all statistical analyses.

## 3. Results

A total of 60 patients were included in the study: 37 patients with MetS and 23 controls. There were no significant differences in age and gender between the groups, while individuals with MetS had significantly more pronounced characteristics of MetS compared to the control group.

In Table 1, we analyzed the ability of high normal values of CRP, the SIRI and our new proposed MetaLGI score to distinguish patients with MetS without metainflammation and those with pronounced metainflammation. Using a threshold of 2.5 mg/L (group 2 vs. 3) for CRP, we showed that patients with pronounced metainflammation (>2.5 mg/L) had significantly higher CRP, fibrinogen, WBC, PLT, ANC and percentage of neutrophils (Table 1a).

On the other hand, an analysis of the SIRI’s ability to separate patients with MetS in relation to metainflammation showed certain differences. Notably, compared to the previous analysis, patients with pronounced metainflammation, indicated by a high SIRI, also had significantly higher CRP, fibrinogen, WBC, ANC, and the percentage of neutrophils, while the percentage of lymphocytes was significantly lower in addition to significantly higher AMC and NLR (Table 1b).

Finally, the proposed MetaLGI score demonstrated superior performance compared to the previous two definitions in distinguishing patients with MetS without metainflammation and those with pronounced metainflammation (Table 1c). Patients with pronounced metainflammation, indicated by a positive MetaLGI score, had higher levels of CRP, WBC, ANC, ALC, AMC, and PLT. Furthermore, the MetaLGI score was superior in detecting higher levels of IL-6 and HOMA-B in patients with MetS and pronounced metainflammation compared to the other two definitions. Additionally, we observed a trend toward a decrease in the number of patients in the subgroup with pronounced metainflammation when comparing various definitions of metainflammation.

**Figure 1:**
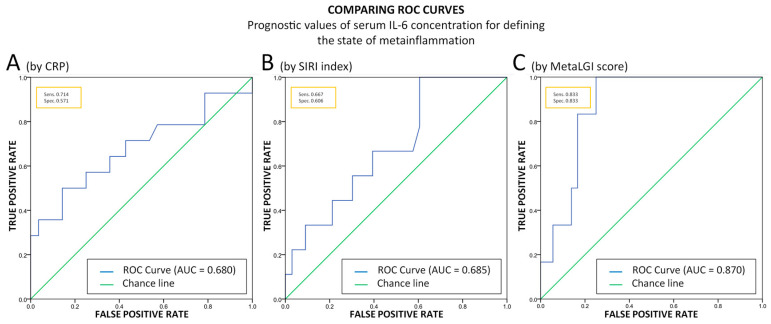
Prognostic values of serum IL-6 using three proposals for defining the state of metainflammation; (**A**) serum CRP concentration; (**B**) SIRI; (**C**) newly proposed MetaLGI score.

The formation of the ROC curve (95%CI) and the calculation of the AUC of IL-6 showed the great advantage of using the MetaLGI score compared to the use of CRP or the SIRI (Figure 1). By comparing groups 1 and 3, our newly proposed score, the MetaLGI score, showed superiority over high normal values of CRP and the SIRI because it showed statistically significantly higher values of all pro-inflammatory parameters, with the simultaneous absence of a decrease in the lymphocyte % and an increase in the NLR in the subgroup with MetS and a positive MetaLGI score compared to the control group of subjects. The proportion of subjects with a positive MetaLGI score was significantly higher in the MetS group than in the control group (24.3% [9/37] vs. 4.3% [1/23], *p* = 0.044).

## 4. Discussion

Until now, the state of chronic metainflammation has been mostly defined by the high normal concentration of normal or high-sensitivity CRP [22]. However, obtaining new data regarding metainflammation and shifting the focus and paradigm towards even earlier detection of pathological process on the continuum of obesity-related diseases requires a more precise, comprehensive, and at the same time inexpensive and widely available approach to the problem [23,24,25]. Our proposed definition aims to address this need by incorporating routinely available laboratory parameters that can be easily integrated into everyday clinical practice across healthcare settings with varying levels of resources, thereby enhancing its practicality, feasibility, and potential for broader clinical application.

The combination of high normal values of inflammatory markers such as CRP (>2.5 mg/L) and fibrinogen (>3.0 g/L), and a high normal number of monocytes (>0.55 × 10^9^) and platelets (>250 × 10^9^), can provide additional insights into the inflammatory and prothrombotic state, confirming the presence of metainflammation and potentially contributing to the overall prothrombogenic assessment of the same state. The dysregulated interaction between inflammation and platelets has the potential to contribute to thrombosis and cardiovascular complications but also events in the brain (stroke) or periphery (peripheral thrombosis) [26]. Considering the mentioned effects of metainflammation on different and, at the same time, related processes of inflammation and thrombosis, our new, more accurate definition of metainflammation would enable a better assessment of the risk of thrombosis in patients with obesity-related diseases, and at the same time further research into the potential effects of metainflammation.

Most current indices predominantly measure only the inflammatory or only the metabolic component on this continuum, while an increasing amount of data clearly speak of the intertwining and interrelations of simultaneous processes of inflammation and metabolic disorders that together clinically lead to atherothrombosis. Considering all of the above, we believe that the new proposal for the definition of metainflammation would be just a starting point and useful tool in assessing the future risk of thrombosis in patients with metabolic syndrome. A negative MetaLGI score means that in that patient we have simultaneously low normal values of CRP, fibrinogen, monocyte and platelet counts, which, considering the whole context of their interrelationships that we gave in the Introduction, reduces the risk of thrombosis compared to people who simultaneously have high-normal or elevated values of CRP, fibrinogen, monocyte and platelet counts. We believe that our proposal will be only the first step in the further development of definitions that can be adapted to other specificities and systems. We also believe that it is time to break scientific inertia on this topic and that it is necessary to talk about it more, even to start a discussion about the need to find a greater number of precise definitions of the state of metainflammation in relation to organic systems, in order to recognize earlier the potential complications that this chronic condition can cause and to work on their prevention.

## 5. Conclusions

Based on all of the above, it seems that the proposal of our new laboratory definition of the state of metainflammation better separates patients with pronounced metainflammation compared to other earlier definitions, which could greatly help in easier recognition and better treatment of this sensitive group of patients before the occurrence of an unwanted cardiovascular event. Given the cross-sectional study design, further longitudinal studies are needed to confirm our hypotheses and results.

## Figures and Tables

**Table 1:** Laboratory parameters in different groups of patients. (**a**) MS patients divided by CRP < 2.5 mg/L and ≥2.5 mg/L; (**b**) divided by SIRI < 1.35 × 10^9^ and ≥1.35 × 10^9^; (**c**) divided by MetaLGI score (CRP > 2.5 mg/L, fibrinogen > 3.0 g/L, monocytes > 0.55 × 10^9^/L, PLT > 250 × 10^9^/L).

(**a**)								
	**1: Controls** **(n = 23)**	**2: MS CRP < 2.5** **(n = 18)**	**3: MS CRP > 2.5** **(n = 19)**	**Test**	***p***	**1 vs. 2**	**1 vs. 3**	**2 vs. 3**
IL-6 (pg/mL)	8.35 ± 3.65	8.20 ± 2.81	11.84 ± 6.05	ANOVA	0.066	1.000	0.980	0.194
CRP (mg/L)	1.28 ± 0.73	1.94 ± 1.41	6.51 ± 3.59	KWtest	0.000 *	0.041	0.000 *	0.000 *
Fibrinogen (g/L)	2.72 ± 0.46	2.95 ± 0.56	3.56 ± 0.56	ANOVA	0.000 *	0.593	0.000 *	0.002 *
WBC (×10^9^/L)	6.57 ± 1.23	6.42 ± 1.28	9.34 ± 2.21	ANOVA	0.000 *	1.000	0.000 *	0.000 *
Neutrophils (%)	54.71 ± 5.47	55.01 ± 9.82	63.25 ± 5.86	KWtest	0.000 *	0.462	0.000 *	0.004 *
Lymphocytes (%)	33.90 ± 5.18	30.40 ± 8.52	26.87 ± 5.26	KWtest	0.002 *	0.198	0.000 *	0.092
Monocytes (%)	8.17 ± 1.61	7.84 ± 1.30	7.00 ± 1.19	ANOVA	0.031 *	1.000	0.029	0.228
ANC (×10^9^/L)	3.41 ± 0.66	3.90 ± 1.33	5.95 ± 1.75	KWtest	0.000 *	0.222	0.000 *	0.000 *
ALC (×10^9^/L)	2.11 ± 0.54	2.06 ± 0.49	2.45 ± 0.59	ANOVA	0.064	1.000	0.155	0.101
AMC (×10^9^/L)	0.50 ± 0.15	0.54 ± 0.23	0.65 ± 0.19	KWtest	0.023 *	0.599	0.008 *	0.049
NLR	1.67 ± 0.42	1.95 ± 0.64	2.23 ± 0.44	KWtest	0.007 *	0.168	0.001 *	0.164
AST (U/L)	22.33 ± 5.55	28.11 ± 8.07	23.63 ± 6.85	ANOVA	0.031 *	0.033	1.000	0.154
ALT (U/L)	17.73 ± 8.46	38.44 ± 17.98	28.63 ± 16.55	KWtest	0.000 *	0.000 *	0.001 *	0.031
PLT (×10^9^/L)	259.26 ± 41.24	229.98 ± 26.64	285.80 ± 63.05	ANOVA	0.002 *	0.145	0.205	0.002 *
HOMA-IR	0.89 ± 0.39	2.63 ± 1.78	2.93 ± 1.76	ANOVA	0.000 *	0.001 *	0.000 *	1.000
HOMA-B	116.73 ± 81.20	116.27 ± 77.99	156.64 ± 123.81	KWtest	0.529	0.946	0.327	0.343
(**b**)								
	**1: Controls** **(n = 23)**	**2: MS SIRI −** **(n = 23)**	**3: MS SIRI +** **(n = 14)**	**Test**	***p***	**1 vs. 2**	**1 vs. 3**	**2 vs. 3**
IL-6 (pg/mL)	8.35 ± 3.65	9.26 ± 4.91	12.22 ± 5.59	ANOVA	0.120	1.000	0.126	0.404
CRP (mg/L)	1.28 ± 0.73	3.28 ± 3.15	5.94 ± 3.71	KWtest	0.000 *	0.001 *	0.000 *	0.008 *
Fibrinogen (g/L)	2.72 ± 0.46	3.06 ± 0.54	3.59 ± 0.65	ANOVA	0.000 *	0.145	0.000 *	0.010 *
WBC (×10^9^/L)	6.57 ± 1.23	6.72 ± 1.44	9.89 ± 2.18	ANOVA	0.000 *	1.000	0.000 *	0.000 *
Neutrophils (%)	54.71 ± 5.47	56.88 ± 5.57	63.11 ± 11.98	KWtest	0.000 *	0.177	0.000 *	0.001 *
Lymphocytes (%)	33.90 ± 5.18	32.39 ± 5.09	22.33 ± 5.52	KWtest	0.000 *	0.307	0.000 *	0.000 *
Monocytes (%)	8.17 ± 1.61	7.26 ± 1.24	7.66 ± 1.39	ANOVA	0.107	0.107	0.915	1.000
ANC (×10^9^/L)	3.41 ± 0.66	3.82 ± 0.93	6.81 ± 1.46	ANOVA	0.000 *	0.480	0.000 *	0.000 *
ALC (×10^9^/L)	2.11 ± 0.54	2.17 ± 0.57	2.42 ± 0.56	ANOVA	0.269	1.000	0.355	0.585
AMC (×10^9^/L)	0.50 ± 0.15	0.48 ± 0.10	0.79 ± 0.22	KWtest	0.000 *	0.991	0.000 *	0.000 *
NLR	1.67 ± 0.42	1.82 ± 0.48	2.62 ± 0.26	KWtest	0.000 *	0.262	0.000 *	0.000 *
AST (U/L)	22.33 ± 5.55	27.30 ± 7.78	23.35 ± 7.18	KWtest	0.061	0.027	0.934	0.094
ALT (U/L)	17.73 ± 8.46	34.73 ± 17.83	31.21 ± 18.01	KWtest	0.000 *	0.000 *	0.000 *	0.298
PLT (×10^9^/L)	259.26 ± 41.24	244.33 ± 39.04	282.15 ± 71.49	ANOVA	0.084	0.921	0.523	0.081
HOMA-IR	0.89 ± 0.39	2.74 ± 1.69	2.86 ± 1.91	KWtest	0.000 *	0.000 *	0.001 *	0.817
HOMA-B	116.73 ± 81.20	129.67 ± 110.65	149.04 ± 96.78	KWtest	0.521	0.982	0.270	0.360
(**c**)								
	**1: Controls** **(n = 23)**	**2: MS SCORE −** **(n = 28)**	**3: MS SCORE +** **(n = 9)**	**Test**	***p***	**1 vs. 2**	**1 vs. 3**	**2 vs. 3**
IL-6 (pg/mL)	8.35 ± 3.65	9.03 ± 4.91	14.36 ± 4.46	KWtest	0.016 *	0.987	0.004 *	0.010 *
CRP (mg/L)	1.28 ± 0.73	3.75 ± 3.73	5.94 ± 2.51	KWtest	0.000 *	0.000 *	0.000 *	0.010 *
Fibrinogen (g/L)	2.72 ± 0.46	3.11 ± 0.62	3.74 ± 0.42	ANOVA	0.000 *	0.039	0.000 *	0.029
WBC (×10^9^/L)	6.57 ± 1.23	7.16 ± 1.89	10.28 ± 1.98	ANOVA	0.000 *	0.439	0.000 *	0.000 *
Neutrophils (%)	54.71 ± 5.47	58.33 ± 9.41	62.07 ± 7.05	KWtest	0.010 *	0.023	0.008 *	0.190
Lymphocytes (%)	33.90 ± 5.18	28.76 ± 7.61	28.05 ± 5.91	ANOVA	0.013 *	0.021	0.080	1.000
Monocytes (%)	8.17 ± 1.61	7.51 ± 1.28	7.11 ± 1.36	KWtest	0.117	0.130	0.094	0.229
ANC (×10^9^/L)	3.41 ± 0.66	4.47 ± 1.68	6.44 ± 1.62	KWtest	0.000 *	0.007 *	0.000 *	0.006 *
ALC (×10^9^/L)	2.11 ± 0.54	2.08 ± 0.44	2.83 ± 0.59	ANOVA	0.001 *	1.000	0.002 *	0.001 *
AMC (×10^9^/L)	0.50 ± 0.15	0.56 ± 0.22	0.71 ± 0.16	KWtest	0.007 *	0.334	0.001 *	0.011 *
NLR	1.67 ± 0.42	2.08 ± 0.57	2.11 ± 0.57	ANOVA	0.017 *	0.023	0.168	1.000
AST (U/L)	22.33 ± 5.55	27.25 ± 8.26	21.33 ± 2.73	ANOVA	0.017 *	0.044	1.000	0.079
ALT (U/L)	17.73 ± 8.46	36.60 ± 19.06	23.44 ± 6.44	KWtest	0.000 *	0.000	0.024	0.023
PLT (×10^9^/L)	259.26 ± 41.24	238.51 ± 39.49	321.28 ± 54.37	KWtest	0.000 *	0.050	0.003 *	0.000 *
HOMA-IR	0.89 ± 0.39	2.78 ± 1.82	2.79 ± 1.61	KWtest	0.000 *	0.000 *	0.000 *	0.804
HOMA-B	116.73 ± 81.20	104.59 ± 70.35	226.80 ± 146.21	KWtest	0.050	0.815	0.041	0.016 *

Abbreviations: ALC—absolute lymphocyte count; AMC—absolute monocyte count; ANC—absolute neutrophil count; ANOVA—one-way analysis of variance; CRP—C-reactive protein; HOMA-B—Homeostasis Model Assessment of Beta-cell function; HOMA-IR—Homeostatic Model Assessment for Insulin Resistance; IL-6—interleukin 6; KWtest—Kruskal–Wallis test; LGI score—low-grade inflammation score; MS—metabolic syndrome; NLR—neutrophil-to-lymphocyte ratio; PLT—platelet count; SIRI—systemic inflammation response index; WBC—white blood cell count. Results are presented as mean ± standard deviation. Note: * is statistically significant.

## Data Availability

The original contributions presented in this study are included in the article. Further inquiries can be directed to the corresponding author.
